# Are Children Harmed by Being Locked up at Home? The Impact of Isolation during the COVID-19 Pandemic on the Phenomenon of Domestic Violence

**DOI:** 10.3390/ijerph192113958

**Published:** 2022-10-27

**Authors:** Jagoda Grzejszczak, Agata Gabryelska, Agnieszka Gmitrowicz, Magdalena Kotlicka-Antczak, Dominik Strzelecki

**Affiliations:** 1 Department of Child and Adolescent Psychiatry, Medical University of Lodz, 90-151 Lodz, Poland; 2 Department of Sleep Medicine and Metabolic Disorders, Medical University of Lodz, 90-151 Lodz, Poland; 3 Department of Affective and Psychotic Disorders, Medical University of Lodz, 90-151 Lodz, Poland

**Keywords:** violence against children and adolescents, COVID-19, neglect, abuse, domestic violence

## Abstract

The phenomenon of violence against children is a very complex one. There are many types of child abuse, and they are culturally dependent to a significant degree. Although studies show that children generally only suffer from mild COVID-19 infection, some social restrictions introduced during the pandemic, such as home isolation, may have many severe consequences on the population’s mental health. Studies on this topic suggest that violence against children increased during lockdown due to the COVID-10 pandemic. This narrative review summarizes this available literature on the subject and discusses the different forms of violence against children, their cultural aspects, the impact of the COVID-19 pandemic on the phenomenon of violence, the long-term consequences of the above, and forms of assistance for abused minors.

## 1. Introduction

Violence against children is a widespread phenomenon. The family remains the most common source of violence in the early years of human life [1].Childhood is a critical period during which one’s personality is formed [2]. Those who do not grow up with healthy relationship models during childhood have difficulty building relationships in the future [3]. Adults who do not provide adequate care to minors expose them to extensive emotional and social consequences and an increased risk of being a victim of future violence [4]. Moreover, children and adolescents exposed to community violence display a broad spectrum of psychological symptoms, ranging from depression and anxiety to antisocial and suicidal behaviors [5].

This narrative review aims to describe the problem of violence against children and adolescents during the COVID-19 pandemic, a period during which victims of violence often constantly remained under the same roof as their aggressor. Additionally, we will discuss this mutilating and dangerous phenomenon and consider solutions that might help reduce its scale.

## 2. Violence in Cultural Terms

The words violence and abuse are used interchangeably. The American Psychiatric Association defines a violent person as one who acts or has acted in such a way as to produce physical or mental harm [6].

The risk of experiencing violence is associated with many important factors, for example, the child’s age, psychosocial context, and cultural background [1]. Violence risk factors can be divided into modifiable and unmodifiable. Among the factors beyond our control, cultural origin occupies a particular role. In many countries, violence against adolescents and children remains the standard and even desirable educational method [7]. The exact breakdown is presented in Table 1.

There are many types of violence—neglect, abuse (physical, verbal, sexual), violence on the Internet, and many others, as will be subsequently elaborated upon [8,9]. Ethnicity plays an important role as far as violent behaviors towards children are concerned. In the cultural context, one of the most controversial seems to be ritual violence, including genital mutilation [10]. One should also remember that children are involved in political or armed conflicts as not only passive but also active participants [11]. Due to ethnic diversity, each continent is characterized by the risk of specific forms of violence against children. Various forms of violence are typical for particular cultural groups.

### 2.1. Europe

According to the World Health Organization (WHO), almost every third child in Europe experiences some form of violence. This means up to approximately 55 million abused children [12]. Even in Denmark, where the welfare system appears to be very sensitive to child abuse, statistics show that approximately 20% of children are affected by physical abuse [13]. Research conducted in different countries indicates various contexts of the phenomenon of violence [14]. Scandinavian studies show that mothers of children with neurological or intellectual disabilities are more likely to resort to violent responses to crises in the educational process [15]. Studies in the Balkan countries highlight the transgenerational transmission of the violent family model [16,17]. Researchers from many countries emphasize the presence of peer violence from an early age [16]. Common peer violence should also be mentioned, including so-called dating violence (DV), bearing the hallmarks of sexual violence. [18,19]. DV, defined as the commission of violence by one or both members of a couple in the context of dating, is a serious public health problem due to its alarming prevalence and damaging effects on the health and wellbeing of young teenage boys and girls [18]. Research has also shown that the definition of violence differs depending on the sex of the responders. Girls describe more behaviors as violent than boys [20]. The armed conflict in Ukraine in recent months is not without significance. Escalating conflict in Ukraine poses an immediate and growing threat to the lives and well-being of the country’s 7.5 million children [21]. This type of violence can lead to mental disorders such as depression and anxiety disorders, e.g., post-traumatic stress disorder [21]. One should try to provide an institutionally stable environment for refugee children in many fields, including education, to minimize the risk of mental disorders [22]. The issue of children who require long-term treatment (internal, oncological, psychiatric) is also important, as psychosocial stress, the uncertainty of the future, and difficulty in collecting existing documentation may make it difficult to provide assistance to war victims in Ukraine [23].

### 2.2. North America

Firearm injury among children and teenagers in the United States remains an area of significant public health concern [24]. The United States is, on the one hand, a culturally diverse country that gives many freedoms and rights to its citizens. On the other hand, statistics show that homicide is the second cause of death for people aged 10–24. Broad access to weapons can be highlighted as one of the causes. The statistics show that almost 60% of children under the age of 17 in the USA suffer from some form of violence, and almost 50% have been attacked at least once with the use of firearms [25,26]. There is a much higher percentage of exposure to firearm injuries among the young Black population. Higher involvement in gang activities, living in neighborhoods with high rates of gun violence, and low household income are among the possible reasons explaining the high Black representation among pediatric firearm injuries [24]. The United States is an ethnically diverse country with a high risk of racist behavior. In 2020, individuals of all ages engaged in demonstrations condemning police brutality and supporting the Black Lives Matter (BLM) movement. Prosocial activities of this type are aimed at minimizing the risk of racial violence [27]. Gangs, human trafficking, drug cartels, and migrations to the north are widely present in the everyday life of the South American population [28]. Metering and “Remain in Mexico” policies force families back to dangerous Mexican border communities where cartels target migrants. Human Rights First has compiled at least 1114 publicly reported cases of murder, rape, torture, kidnapping, and other acts of violence against asylum seekers in the Remain in Mexico Program, including 265 children who were kidnapped or nearly kidnapped [28]. This means nearly 200 million abused children. The effects of traumatic experiences such as being left unattended or witnessing experiences will possibly have a severe impact on their future mental health [26,28]. Interesting research focusing on the potential relationship between commitment to a religious group and the risk of exposure to violence was conducted in Canada. Data on this phenomenon are not consistent. However, a disturbing trend may indicate an increased risk of exposure to violence in a group of religiously engaged people compared to those who do not belong to such communities [29].

### 2.3. South America

Latin American countries are also highly ethnically diverse, particularly in the cases of the populations of Brazil, Colombia, and Venezuela. The official discourse points to several protective measures concerning ethnic minorities. However, research shows that violence against children appears in many different forms—neglect, sex trafficking of teenage girls, participation of minors in drug gang activities, forced physical labor, and corporal punishment [30]. Other studies examining the prevalence of physical violence among three thousand Haitians aged 13–24 show that two-thirds experienced physical violence during childhood. Most of them, regarding each area, experienced violence from parents or teachers who intended to punish or discipline their children [31]. In Haiti, as in many developing countries, the phenomenon of street children remains a serious problem. Research shows that street children experience multiple traumas, such as neglect, maltreatment, and psychological, physical, and sexual abuse [32]. Consideration should also be given to the child population of Brazil, where neglect is the main form of violent behavior [33]. It should be emphasized that data for South America are often incomplete and statistics underestimated [34].

### 2.4. Asia and Pacific Region

There is a disproportionate difference between Asia and the Pacific region, however, the statistics regarding each area are worrying. It has been estimated that 8 million children are affected by any form of violence in Oceania, and over a billion children experience violence in the Asian continent [26,35]. A study in East Asia and the Pacific region found that the cost of violence against children amounts to 2% of its gross domestic product (GDP) [35]. In many countries in this region, the patriarchal family system is predominant. Domestic violence against women and girls is a common phenomenon in this area [36]. The burden of widespread armed conflicts in this region should also be mentioned. Recently, minors affected by the Palestinian armed conflict has been an important issue [37]. Children not only participate in fights but also become refugees, which prevents or at least significantly hinders their further development [38]. Other studies emphasize that physical, sexual, or emotional violence against adolescents is highly prevalent here and that many teenagers—young wives—are victims of partner violence [39,40]. There is a large child workforce in India, estimated at 40 million—comprising one-fifth of the world’s total child population [41]. Child labor is considered a modern form of slavery. Children have no right to object. Poverty and illiteracy are the root causes of this phenomenon. Children are employed in various professions—agriculture, domestic labor, and the automotive industry. They are at risk of health loss and even threats to their life. Child trafficking and sexual slavery are also significant [42].

Research on forms of violence in China and South Korea focuses on violence by parents and teachers. Interesting research comparing the child populations of China and South Korea indicates that violence by both parents and teachers is more common in South Korea. It can be hypothesized that this results from, for example, the much higher level of China’s economic development [43].

### 2.5. Africa

Data collected in 2015 in South Africa indicate that the value of children disability-adjusted life years (DALYs)—an indicator used to determine the health condition of a given society—lost to violence as approximately 4% of South Africa’s GDP from that year [44]. The number of children affected by violence at that time was also terrifying. Half a billion children have been affected by any form of violence [26]. The Kenyan Democratic and Health Survey estimated that 21% of women aged 15–49 in this country had experienced female genital mutilation [45]. In low-income countries, apart from the cultural context, the economic factor is the key factor influencing the scale of violence [44,46]. Inability to provide the basic needs of a typically large family in poor African regions increases frustration, which can result in resorting to unacceptable educational methods [47]. Furthermore, compared to Europe, the prevalence of violence against children with disabilities is greater [48]. Culturally, violence is used as an instrument of discipline, as in other continents, mainly in low-income countries [49].

## 3. Violence during the COVID-19 Pandemic

The world faces various cyclical global difficulties. Human beings are largely to blame for the climate catastrophe, hunger and malnutrition, armed conflicts, oppressive political and cultural systems, and, finally, pandemics. Children, teenagers, women, and the elderly are usually the most vulnerable in these situations. Incidences of domestic violence, child abuse, and adulterated online content are rising [50]. Recently, the world has experienced an enormous burden—the COVID-19 pandemic. As predicted, this global health, economic, and social crisis associated with the pandemic may affect the next few generations [51]. The full extent of the consequences of this experience remain unknown. However, today we can say that the pandemic has evidenced significant gaps in many countries’ economic systems.

UNICEF research from 2014 showed that 68.5% of children in Egypt have experienced any type of violence [52]. In April 2020, an online survey among parents of children under 18 was conducted in Egypt to determine whether the percentage of violence during the pandemic changed. The results show that out of 1118 completed survey responses, 90.5% of children were subjected to violent discipline, 88.7% experienced psychological aggression, and 43.2% encountered severe physical punishment. Approximately 60% of respondents reported a moderate-to-severe psychological impact (assessed by Impact of Event Scale—Revised (IES-R) scores ≥ 33; a result indicating an increased risk of PTSD), which was associated with a higher rate of violent discipline [53]. In Jianli County (Hubei province, China), incidences of domestic violence reported to the police tripled during the lockdown in February—from 47 in 2019 to 162 in 2020 [54]. Home isolation during the pandemic proved to be a significant challenge for families. Being constantly enclosed in flats contributed to increased tension between family members [55]. Many adults feared both the SARS-CoV-2 virus infection and losing their jobs [56]. Children were deprived of contact with peers and very often deprived of adequate mental support [57]. Spending many days together in a small space has previously been known to aggravate smoldering conflicts [58]. Fabbri et al., in their study estimating the anticipated effect of COVID-19 on violent discipline against 1–14-year-old children at home, reported that in Nigeria, Mongolia, and Suriname, under a “high restrictions” scenario, there would be a 35–46% increase in violent discipline scores, and under a “lower restrictions” scenario, there would be only a 4–6% increase [59]. US researchers found that firearm violence also increased in scale during the COVID-19 pandemic. There were 1076 child-involved shootings in 2020, 811 in 2019, and 803 in 2018. From 2018 to 2020, the data show an evident increase in incidences of children killed by adults [60].

Research indicates that isolation exacerbated pre-existing mental disorders in children and adolescents. The most common symptoms were anxiety and depression [61]. Due to the global scale of the problem caused by the COVID-19 pandemic, many studies focusing on mental health also evaluated domestic violence. Anxiety and insecurity among adults further increased the risk of violence against children. Research has shown an apparent increased use of helplines during the recent crisis [52,62]. For example, Canada’s Kids Help Phone saw a 112% increase in calls in April 2020 compared to the previous year, which included a spike of 28% for calls specifically related to violence [52]. Cutting off direct contact with peers also cuts off natural support. The situation of many young people under the care of psychiatrists or psychotherapists before the pandemic has also changed [63]. During lockdown, the popularity of telemedicine significantly increased. Consequently, this made the Internet and social media areas of indirect therapeutic contact [64]. As a pandemic also poses a risk of severe illness and death, many children also recently lost one or two guardians [65]. Thus, they are exposed to poverty, violence, and institutionalization [51]. Regardless of socio-economic conditions, research highlights an increase in reports of sexual violence against women, including women under the age of 18 [66].

For example, a 61.6% increase in child abuse disclosures during the COVID-19 pandemic was reported in South Africa compared to the previous year. Emotional abuse was the most frequent, followed by physical and sexual abuse [45]. This staggering statistic highlights the need for various societies to modify their welfare systems to minimize the risk of violence against youth in the future, especially in the event of another global crisis [67]. Studies have shown that the level of violence increased during the COVID-19 pandemic [68,69]. In the US, the severity of domestic violence was assessed using geolocators [70]. Due to an increase in the frequency of family members working from home, the percentage of domestic violence incidence in the US is estimated to have increased from 12 to 20% in the US during the isolation caused by the COVID-19 pandemic. The number of first incidents of domestic violence incidence also increased from 16 to 23%. Studies performed in South Korea and North Carolina (USA) have shown a link between a parent’s job loss and a significant increase in the risk of child abuse [71,72]. This highlights the contribution of stress caused by fanatical instability and the presence of a parent at home during the day to possible child abuse. On the other hand, in the Netherlands, the occurrence of possible changes in the phenomenon of violence in families already affected by this problem was investigated. No progression or regression in family violence was observed, suggesting that an increase may be observed mainly due to new cases rather than an increase in already present violence [73]. The studies compiled thus far show a noticeable increase in the phenomenon of violence against children on various continents. It is difficult to frame it in a cultural context, as this aspect has not been assessed as a possible risk factor. Additionally, further observations are necessary to evaluate the long-term effects of COVID-19 on the phenomenon of violence [74]. Much of the research conducted to date evaluated violence in general, as data specific to the child population are still very limited, and some risk factors and cultural contexts remain partly unknown.

## 4. Numerous Consequences

**Figure 1:**
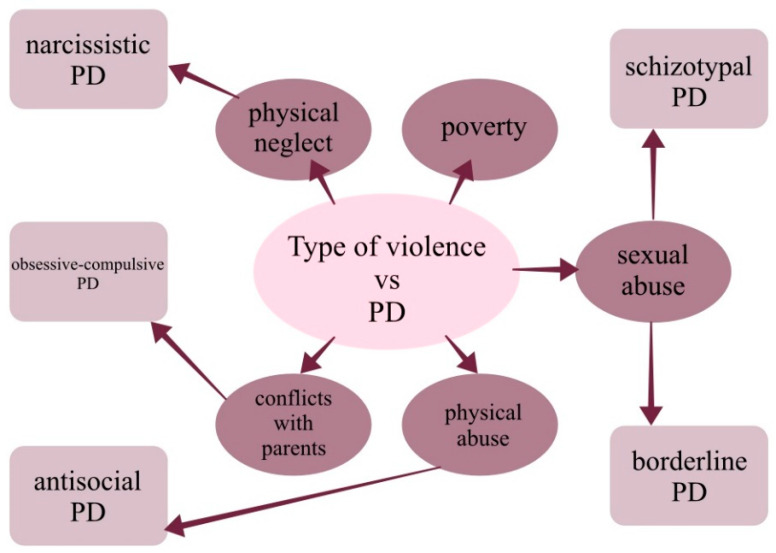
Type of childhood violence vs. type of personality disorder that may occur in adulthood. Abbreviations: PD—personality disorder.

Childhood is the time when one’s mental construction is being formed. The experience of violence has a direct impact on this. Violence affects one’s physical, mental, and social health [2]. For their favorable development, children require a stable and safe environment. When domestic violence occurs, the balance of the family system is disturbed, and all its members suffer the consequences of the violence. More than one billion children—half of all children worldwide—are exposed to violence every year [75]. Victims of violence will carry the scar of violence throughout their lives. Hitting a child has emotional, social, and economic consequences [75,76]. Child mistreatment remains a major public health and social welfare problem in high-income countries. Every year, 4–16% of children are physically abused, and one in ten is neglected or psychologically abused. Between 5% and 10% of girls and up to 5% of boys are exposed to penetrative sexual abuse during their childhood, and up to three times more children are exposed to any type of sexual abuse [77]. Studies addressing the biological underpinnings of such consequences demonstrate that violence-associated stress may cause damage to the nervous, endocrine, circulatory, musculoskeletal, reproductive, respiratory, and immune systems [75]. The most common forms of domestic violence are physical and emotional abuse in the presence of violence against one’s mother and, to a lesser extent, sexual abuse. In addition, physical, emotional, educational, and medical neglect remain significant problems in many societies [76]. The presence of violence against one’s mother and the feeling of impotence experienced by the child may leave the same consequences as the endured violence. It is considered that children living in violent families are likely to live under cumulative stress [78]. This carries an increased risk of depression, anxiety, and a greater risk of suicide [79,80]. An important issue is also the impact of the exposure to childhood violence on the occurrence of personality disorders (PDs) in adult life. The analysis provided by Hengartner et al. revealed that all PD dimensions were significantly related to various forms of previous family and school problems and child abuse. In contrast, according to the multivariate analysis, only school problems and emotional abuse were associated with various PDs. Poverty was uniquely related to schizotypal PDs, conflicts with parents with obsessive compulsive PD, physical abuse with antisocial PDs, and physical neglect with narcissistic PDs. Sexual abuse was statistically significantly associated with schizotypal and borderline PDs. Bullying and violence in schools and emotional abuse appear to be more salient markers of general personality pathology than other forms of childhood adversity (Figure 1) [81].

Responses to traumas include a wide range of conditions, from acute stress reactions through post-traumatic stress disorders to complex, long-lasting, repeated trauma syndrome [78]. It has been shown that children having experienced violence show significant deficits in building healthy relationships [76,82]. Child maltreatment substantially contributes to drug and alcohol misuse (especially in girls), risky sexual behavior, and criminal behavior, which persist into adulthood [77].

Violence by teachers in schools is not without significance. However, there is not much research on this subject. It is noted that children who experience any type of violence at school may develop reactive attachment disorder, modest physical inactivity, overweight or obesity, diabetes, smoking habits, heavy alcohol use, poor self-rated health, cancer, heart disease, and respiratory disease, and as well as other negative outcomes [83].

Children who are victims of global conflicts, war, terrorism, catastrophes, or humanitarian crises also need to be mentioned [84,85,86]. Research shows the potential for the lasting adverse effects of such childhood experiences, possibly making individuals more vulnerable as adults [86]. In his review, Williams draws attention to publications that focus on children and young people as victims of violence and what psychosocial impacts of death, divorce, accidents, and disaster they may suffer from [86]. Not fully explored areas include the relationship between childhood violence and eating disorders in adulthood [87].

## 5. State Tools against Violence-Institutional Help

Can we prevent violence against children? The phenomenon is increasingly recognized as a crucial and urgent public health, social, and human rights issue cutting across geographical, socioeconomic, and cultural boundaries. There is no doubt that children need to be protected. However, this is not a task for an individual [88,89]. There is a need for joint, international action on a large scale [90]. Strategies for preventing violence against children have evolved over the last three decades to incorporate community-engaged approaches. These promising approaches involve mobilizing key stakeholders within communities from various sectors and engaging with adult bystanders to take action when violence is suspected. However, many challenges are associated with funding and evaluating such programs, which are often barriers to developing an evidence base that includes metrics of effectiveness and cost benefits [91]. Attention should be paid to the dissonance between countries resulting from their economic status. The description of the phenomenon and the available aid tools are much more extensive in high-income countries compared to low- and middle-income countries [92,93]. As mentioned earlier, the definition of violence is also a problem. It may differ depending on the country. This is mainly due to cultural influences [94]. Despite this, violence against children occurs in all countries, affecting children of all ages, genders, races, and socio-economic strata [88]. The WHO, with partners, created INSPIRE, a comprehensive program of policies, programs, and practices with an integrated approach to violence prevention and response coordinated across the formal and informal settings of civil and private society [95]. INSPIRE takes an integrated approach coordinated across formal and informal settings of civil and private society. The program is based on four strategies: positive parenting, necessities, formal social support, and school structural support, alongside direct and indirect community violence outcomes. Research has shown that children who used more counter strategies were less likely to experience violence [95]. A Special Symposium was organized during the 2018 International Conference on Realistic and Agile Ideas for Children Equality (VOICE) in Bali, Indonesia. With over 250 participants from 23 countries, the conference included 52 presentations and a multi-day learning series on social norms concerning violence against children. The report from the Special Symposium illustrates the importance of social norms in preventing violence against children and the importance of understanding norms in context. The authors emphasized the need to understand how geographic location, social cohesion, group roles and identities, age, and gendered expectations inform whether, when, and which children will experience violence, who perpetrates it, and how individuals and communities respond to it [88]. Despite the difficulties and complexity of the issue, international specialists working for children take up the challenge. Optimal ways of preventing the effects of violence against children are still being sought. It has to be stated that based on collected information, prevention is a critical task in reducing violence against children [96].

## 6. What Else Can We Do?

Sensitivity to harm and the awareness of salient evidence of violence among professionals who work with children seem to be invaluable first steps in providing help for violated children. Global actions are valuable, but it is often a doctor, nurse, social worker, or teacher that is the first rescuer for abused children [97]. The abilities of professionals to establish contact and build trust with a young person are essential [98]. In this regard, continuous training is needed to increase the qualifications of healthcare workers, psychologists, and educators, making them qualified to provide support for abused children. Emphasis should also be placed on educating parents. In abusive families, the phenomenon of unifying secrecy (about abuse, especially sexual abuse) occurs, holding the family system together [99]. Series of media campaigns with a psychoeducational message on the topic is one of the ways to highlight the problem by allowing the victim to speak out. The COVID-19 pandemic has highlighted the importance of helplines [62]. Every effort should be made to increase the number of such forms of support.

## 7. From Theory to Practice

Action to protect children should be continuous and independent of the COVID-19 pandemic. Speaking out or standing up for the vulnerable is an invaluable attribute. Adults should not be afraid to denounce instances of child abuse [100]. One should be open to other cultures but not blindly follow certain ritual (including mutilation) or the cult of patriarchal violence against girls. Patience, understanding, peace, and support are what children need most from adults [101]. The social role of a responsible citizen obliges one to not remain passive in the face of harm [102]. Finally, a victim’s perspective must be considered. Research conducted in Australia evaluated forgiveness as a useful strategy for many victims of various forms of abuse and trauma. Forgiveness is suggested as an effective coping response for ameliorating the aggressive affective states of victimized youth, with further exploration needed regarding the interplay between the avoidance and forgiveness processes [103].

To reduce the multiple serious effects of violence against children, emphasis should be placed on interventions in the family system. According to research, several preventive educational programs for parents bring noticeable results in reducing violence against children. Such parenting programs can effectively reduce physical and emotional violence and the neglect of adolescents, increase parents’ capacity to protect children from sexual violence, and reduce their support for child marriage (controversially, most often not culturally classified as violence) [5]. Competent multidisciplinary teams are needed at national levels to both detect signs of childhood violence early and help combat its long-term effects. Research shows that while, in high-income countries, such teams work effectively, in low- and middle-income countries, there are still many gaps in the systems that need to be improved in the long run [104].

## 8. Conclusions

While violence against children seems to be a thoroughly and holistically researched aspect, violence in the context of the COVID-19 pandemic is a new phenomenon. We are not able to estimate the full extent of the long-term effects of the pandemic crisis. While social isolation during the rapid spread of the coronavirus around the world seems to be the right decision for epidemiological reasons, it is not the optimal solution for mental health. The abrupt withdrawal from school, social life, and outdoor activities greatly impacted children and adolescents. Some of them also experienced a growth in domestic violence. The stress they were subjected can directly affect their mental health because of increased anxiety, lowered mood, and changes in their diets and school dynamics. The full consequences are not yet predictable [105]. It is difficult to generalize the scale of the problem of the increased risk of domestic violence against children during home isolation because many countries used various preventive measures. It seems evident that anxiety for many reasons generated in the family system, feeling of insecurity, and a high level of frustration exacerbate domestic violence. Many aspects of life moved to the Internet during the pandemic, On the one hand, it created a new field for appealing for help in difficult situations, but on the other, it gave rise to new forms of violence. Given the mental health implications of family violence, mental health professionals need to be aware of this issue during the pandemic and ready to assist with developing strategies to overcome the situation. In order to support different social groups at risk of mental well-being imbalance, large-scale action should be taken, bearing in mind that bottom-up action is often the first response to a problem [106].

Much more intensive research is needed during and following the COVID-19 pandemic, with further longitudinal studies to better understand the phenomenon of violence against children in the context of pandemic restrictions and their long-term consequences. This could allow for the avoidance of mistakes and better support for the young generation in a potential similar future global crisis. Research shows an apparent connection between childhood violence and intimate partner violence (IPV). Interventions aimed at promoting healthy relationships and providing emotional support and coping mechanisms to children and families in abusive situations are key components to ending the cycle of violence and preventing IPV in adulthood. Hence, it is vital for future generations to undertake preventive interventions in the present [82].

## Figures and Tables

**Table 1:** Risk factors for violence.

Modifiable Factors	Non-Modifiable Factors
intimate partner violence in parent’s relationship weak family community separation from parents educational model in a family (e.g., corporal punishment) substance abuse in a family family members committing crime media violence exposure peer group school as a source of violence contact with a criminal world week social ties substance abuse in peer group	age male sex country of birth, the economic situation of the state family of birth abused parents generational, cultural transmission separation from parents insufficient parents’ involvement stay in a care facility crime experience biological mental disorders (e.g., attention deficit hyperactivity disorder (ADHD)) low level of intellect being an unplanned child
